# Exploring the association between voice biomarkers, psychological stress and disease severity in atopic dermatitis: A 12‐week decentralized study using patients’ own smartphones

**DOI:** 10.1111/srt.13226

**Published:** 2022-10-30

**Authors:** Zarqa Ali, Teske Valk, Ari Isberg, Mischa Szpirt, Ana Maria Dutei, Simon Francis Thomsen, Aleksander Eiken, Johan Allerup, Theis Bjerre‐Christensen, Miron Derchansky, Anders Daniel Andersen, John Zibert

**Affiliations:** ^1^ Department of Dermato‐Venereology and Wound Healing Centre Copenhagen University Hospital Bispebjerg Copenhagen Denmark; ^2^ Studies&Me A/S Denmark; ^3^ Department of Biomedical Sciences University of Copenhagen Copenhagen Denmark; ^4^ Orthopedic Surgery Nordsjællands Hospital Hillerød Denmark; ^5^ Blackletter Consulting Inc. Toronto Canada

To the Editor:

Several external and internal triggers may cause a flare up of atopic dermatitis (AD), and psychological stress is well known to have an impact on AD disease activity. Stress levels are either self‐reported or require complicated monitoring setups of pulse, blood pressure, and hormone levels. Research into voice analysis has revealed that human emotions, such as stress, can be extrapolated from voice biomarkers exploiting that psychological states alter the characteristics of one's voice.^1^ In this 12‐week decentralized longitudinal study, we aimed to explore the association between selected voice biomarkers and measures of objective and subjective AD disease activity.

Adults with AD fulfilling the UK Diagnostic Criteria and not using any systemic treatment or light therapy were recruited online. All study related activities throughout the 12‐week period were conducted using the patients’ own smartphones or desktop.

Once a week (Sunday) patients were prompted to use their smartphone to photograph up to three self‐selected AD lesions, with a photo capture application (Imagine, LEO Innovation Lab, Denmark), and complete an online questionnaire including Skindex Mini questionnaire,^2^ a numeric ranking scale (NRS) for self‐perceived daily stress, and the patient‐oriented eczema measure (POEM).^3^ Further, patients were asked to perform a 59‐s voice recording with their own mobile device. A layered voice analysis platform (Nemesysco, Israel) was used to extract the sub‐acoustic/low‐amplitude features from the recorded voice samples. All photographs were evaluated by a board‐certified dermatologist. Each photograph was scored according to the intensity part of SCORing Atopic Dermatitis (SCORAD) as per the presence and intensity of the following characteristics, erythema, oozing/crusting, excoriations, lichenification, swelling and xerosis, resulting in an iSCORAD, as previously described^4^, ^5^ (further details are provided in Supporting Information).

Of 141 screened patients, 45 eligible signed the e‐consent, of which 26 completed the study and were included in the final analysis (Figure S1). Baseline characteristics are given in Table S1.

Clear fluctuations in POEM and stress‐NRS were observed, whereas this was not to the same degree evident for iSCORAD (Figure 1). Stress‐NRS was correlated with POEM (*r* = 0.48, 95% confidence interval [CI]: 0.26–0.68, *p* < 0.0001) and iSCORAD (*r* = 0.05, 95% CI: 0.00–0.10, *p* = 0.056).

**FIGURE 1:**
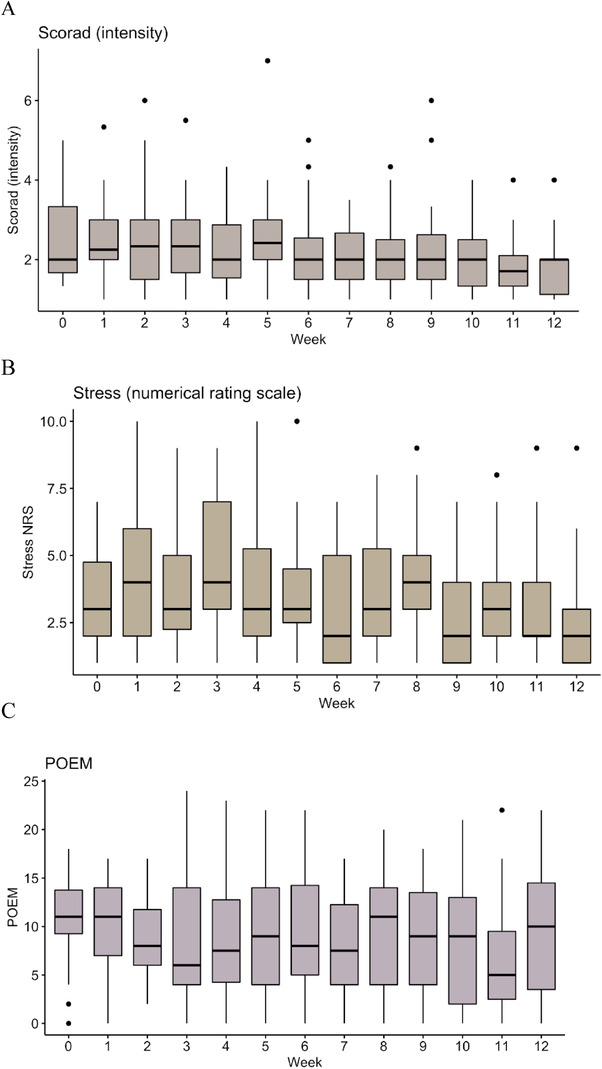
SCORing Atopic Dermatitis (SCORAD) intensity scores (A), stress‐numerical rating scale (B), and patient‐oriented eczema measure (POEM) (C) with median and interquartile range for each week during the 12‐week study period. The points represent single observations.

Even though the voice feature and the AD severity measured as POEM and iSCORAD showed covarying patterns for some patients (Figure 2), a mixed effects model found no statistically significant associations between iSCORAD or stress‐NRS with any of the acoustic features (Table S2).

**FIGURE 2:**
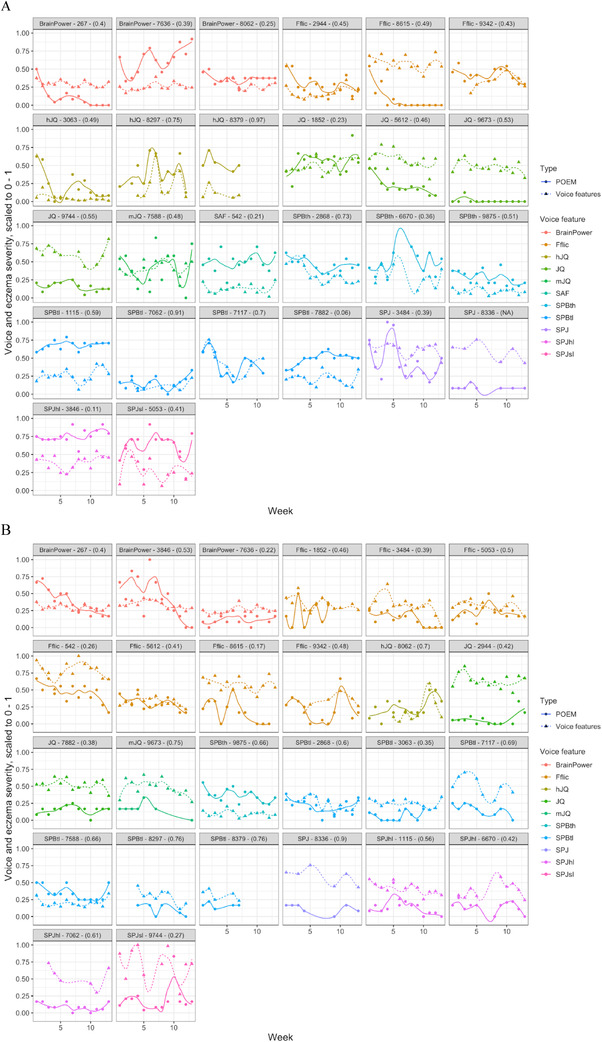
Longitudinal view of patient‐oriented eczema measure (POEM) (A) and iSCORAD (B) for each patient with the highest correlated voice feature. Triangle‐shaped data points with dashed line represent the voice feature, and the color illustrates the particular voice feature. Both variables have been scaled to 0–1 for comparison.

This is to our knowledge the first study investigating the association between voice features and a dermatological condition. A correlation between the iSCORAD and POEM and stress‐NRS was demonstrated, though an overall statistically significant correlation between voice biomarkers and the stress‐NRS, iSCORAD or POEM was not identified. However, at the individual patient level, covarying patterns between voice biomarkers and AD severity were identified. Further studies with bigger sample size are warranted to explore the association between voice biomarkers and AD severity.

## CONFLICT OF INTEREST

TV, AI, MS, AMD, AE, JA, TBC, ADA and JZ are employed by Studies&Me. All other authors have no conflict of interest.

## FUNDING INFORMTION

Studies&Me

## Supporting information

Supporting Information

## Data Availability

The data that support the findings of this study are available on request from the corresponding author. The data are not publicly available due to privacy or ethical restrictions.
