# Supplementary material for: Exploring the association between voice biomarkers, psychological stress and disease severity in atopic dermatitis: A 12‐week decentralized study using patients’ own smartphones
Source: Skin Res Technol. 2022 Oct 30;28(6):882–5. doi: 10.1111/srt.13226 (PMC9907703; doi:10.1111/srt.13226)
Supplement: Supplementary file 1 — Supporting Information [file SRT-28-882-s001.docx]

**Supplementary material**

Inclusion criteria:

1. Age above 18 years or older
2. Fulfilling the UK Diagnostic Criteria

Exclusion criteria

1. Concomitant presence of other skin diseases than atopic dermatitis (AD)
2. Use of systemic medication (including light therapy)
3. Inability of speaking and understanding Danish.

Eligible patients received an email, which contained a link to an online consent to participation prior to study initiation. After enrollment, all patients downloaded a photo capture application onto their smartphones (Imagine, LEO Innovation Lab, Denmark) designed to help patients obtain standardized photographs of skin lesions.

Weekly on Sundays, patients were prompted to photograph up to three self-selected AD lesions, with the option to photograph additional areas if AD occurred outside the target lesions during the study period. If no lesions were visible at the time of inclusion, patients were instructed to select the areas where their AD most often occurred as the target lesions. To optimize photo quality, standardized text, photographic and video material was developed with instruction on how to optimally photograph skin lesions, and sent to the patients after enrolment, prior to the first photograph.

All photographs were evaluated by a board-certified dermatologist using a proprietary tool consisting of a web-based dashboard allowing the dermatologist to view and rate all the photographs one by one.

Patients were asked to carry out a 59 second voice recording with their own mobile device. There were no restrictions on the content of the recording, but to facilitate a proper voice sampling, participants were asked to talk about experiences from the preceding week.

To minimize non-adherence, a two-step reminder system through both email and text messages was established. In cases where a participant had not completed one or more of the weekly study tasks, the participants would receive a reminder to do so the following day (Monday), and then again on Tuesdays, if necessary.

Statistical analysis:

The relationship between POEM, iSCORAD, Stress NRS and the voice features, was analysed using a series of linear mixed effects regression models. This regression method captures both the inter-individual variation and the variation between participants over time. Normality and homoscedasticity was confirmed through visual inspections of Q-Q (quantile-quantile) and residual plots after fitting the linear mixed effects models by a restricted maximum likelihood with the lmer function from the *lme4* R package**.** The 95% confidence intervals were estimated with parametric bootstrapping with 500 replicates.

Ethical approval:

The Danish ethical committee has been informed about the study and evaluated that an ethical approval is not required as the study was purely observational. The data handling of patient sensitive data was approved by the Danish Data Protection Agency and compliant with General Data Protection Regulation.

**Supplementary Figure 1.** Flow chart of the study.


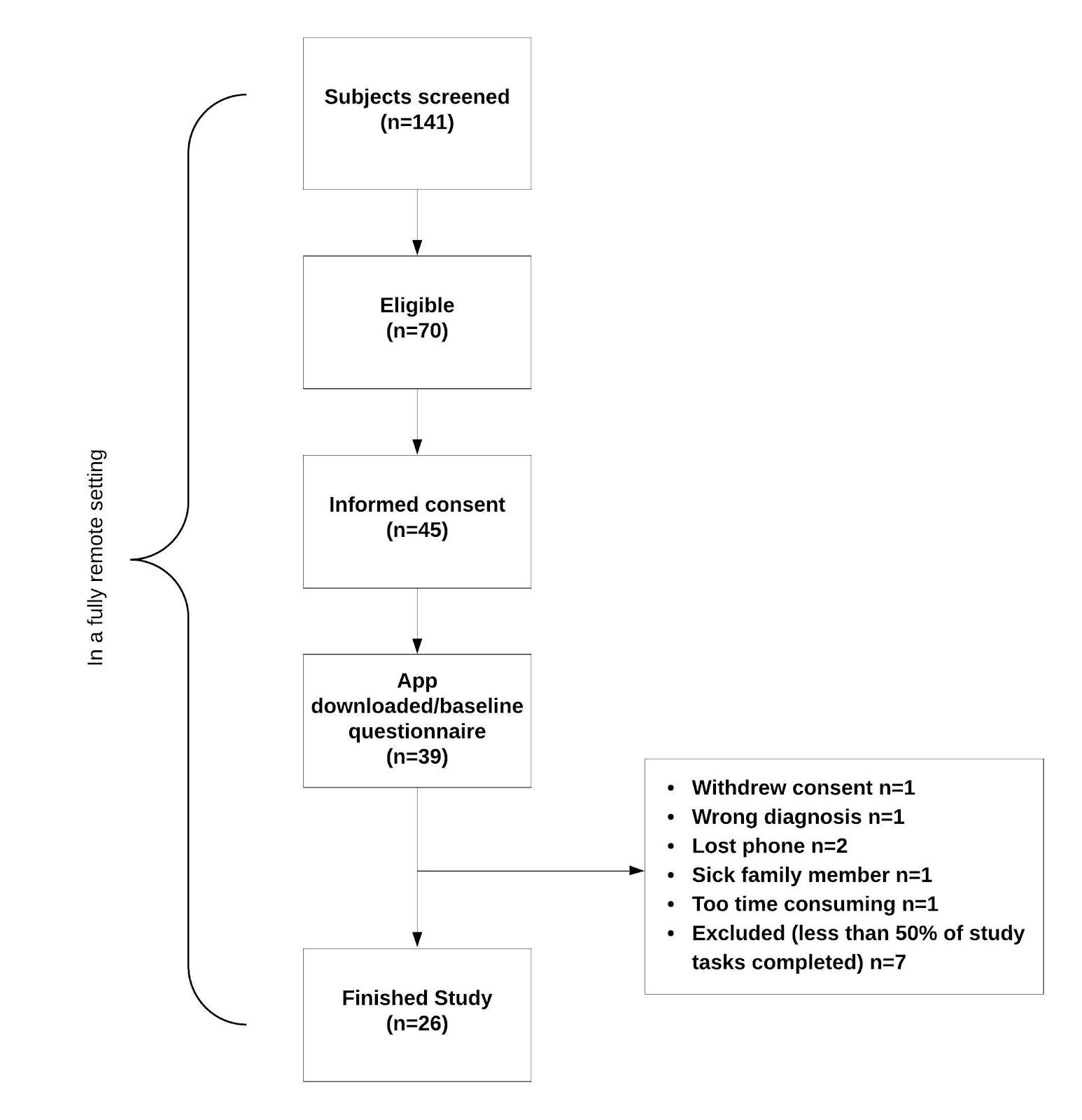


**Supplementary Table 1.** Demographics and characteristics of study participants (n = 26).

| **Sociodemographic data** |  |
| --- | --- |
| Age, years, mean (SD) | 25.5 (5.2) |
| Gender, female, n (%) | 21 (80.7) |
| Cell phone, iOS, n (%) | 15 (57.7) |
| Cell phone, Android, n (%) | 11 (42.3) |
| **Disease severity at inclusion** |  |
| iSCORAD, mean (SD) | 2.6 (1.2) |
| POEM, mean, (SD) | 10.8 (4.6) |
| POEM, Almost clear (0-2), n (%) | 2 (8) |
| POEM, Mild eczema (3-7), n (%) | 3 (12) |
| POEM, Moderate eczema (8-16), n (%) | 19 (73) |
| POEM, Severe eczema (17-24), n (%) | 2 (8) |
| POEM, Very severe (25-28), n (%) | 0 (0) |
| **Yearly flare-ups, self reported** |  |
| 1-5, n (%) | 7 (26.9) |
| 6-12, n (%) | 4 (15.4) |
| all the time (chronic), n (%) | 15 (57.7) |
| **Self reported body surface area** |  |
| 1-3%, n (%) | 15 (57.7) |
| 4-10%, n (%) | 8 (30.8) |
| 11-20%, n (%) | 2 (7.7) |
| 21-40, n (%) | 1 (3.8) |
| **Comorbidities** |  |
| Asthma, n (%) | 8 (30.8) |
| Hay fever, n (%) | 15 (57.7) |
| **Medication** |  |
| No treatment, n (%) | 2 (7.7) |
| Moisturizing cream, n (%) | 2 (7.7) |
| Hormonal cream or similar, n (%) | 2 (7.7) |
| Hormonal and moisturizing cream, n (%) | 20 (76.9) |

**Supplementary Table 2.** Correlations between voice features related to stress and subjective AD severity (POEM), objective AD severity (iSCORAD), as well as self-perceived stress (Stress NRS).

|  | Voice feature | Coefficient | 95% Confidence interval | | Random effect (SD) |
| --- | --- | --- | --- | --- | --- |
|  |  |  | **Lower** | **Upper** |  |
| POEM  Scale: 0-28 | SPJ | 1.27 | -0.751 | 3.46 | 4.78 |
|  | SPJhl | 0.0169 | -0.662 | 0.738 | 4.72 |
|  | SPJsl | 0.22 | -0.0396 | 0.466 | 4.71 |
|  | SPBtl | 0.0363 | -0.0561 | 0.137 | 4.69 |
|  | SPBtl_DIF | 0.0577 | -0.0882 | 0.195 | 4.68 |
|  | SPBth_DIF | -0.055 | -0.139 | 0.0253 | 4.78 |
|  | mJQ | 0.0383 | -0.00807 | 0.0885 | 4.78 |
|  | hJQ | -0.00535 | -0.114 | 0.113 | 4.71 |
|  | JQ | 0.0863 | -0.03 | 0.209 | 4.74 |
|  | SAF | -0.407 | -0.702 | -0.0965 | 4.58 |
|  | BrainPower | 0.23 | -0.317 | 0.766 | 4.64 |
|  | Fflic | 0.506 | -0.572 | 1.51 | 4.77 |
| iSCORAD  Scale: 0-18 | SPJ | -0.000432 | -0.00546 | 0.00462 | 0.656 |
|  | SPJhl | 0.00152 | -0.0147 | 0.0181 | 0.655 |
|  | SPJsl | -0.00308 | -0.00947 | 0.00287 | 0.644 |
|  | SPBtl | -0.000323 | -0.00255 | 0.00176 | 0.653 |
|  | SPBtl_DIF | -0.00319 | -0.0368 | 0.0292 | 0.655 |
|  | SPBth_DIF | 0.00409 | -0.0147 | 0.022 | 0.66 |
|  | mJQ | -0.000275 | -0.0114 | 0.0114 | 0.658 |
|  | hJQ | -0.0013 | -0.0239 | 0.0264 | 0.659 |
|  | JQ | -0.00191 | -0.0279 | 0.0252 | 0.661 |
|  | SAF | -0.0252 | -0.0919 | 0.0425 | 0.657 |
|  | BrainPower | 0.000435 | -0.000725 | 0.00154 | 0.671 |
|  | Fflic | 0.206 | -0.0119 | 0.43 | 0.62 |
| Stress NRS  Scale: 0-10 | SPJ | -0.00393 | -0.0149 | 0.00583 | 1.01 |
|  | SPJhl | -0.0293 | -0.0622 | 0.00528 | 0.911 |
|  | SPJsl | 0.00466 | -0.00811 | 0.0175 | 1.01 |
|  | SPBtl | 0.0019 | -0.00278 | 0.00675 | 1 |
|  | SPBtl_DIF | 0.0356 | -0.0418 | 0.104 | 0.997 |
|  | SPBth_DIF | 0.0237 | -0.0201 | 0.0664 | 0.988 |
|  | mJQ | -0.0162 | -0.0421 | 0.00818 | 0.988 |
|  | hJQ | -0.0505 | -0.1 | 0.00395 | 0.967 |
|  | JQ | -0.00423 | -0.0652 | 0.0552 | 1.03 |
|  | SAF | 0.0438 | -0.103 | 0.186 | 1.05 |
|  | BrainPower | 0.000821 | -0.0018 | 0.00365 | 0.98 |
|  | Fflic | -0.13 | -0.597 | 0.34 | 1.01 |
